# Efficient Sensor Placement Optimization for Shape Deformation Sensing of Antenna Structures with Fiber Bragg Grating Strain Sensors

**DOI:** 10.3390/s18082481

**Published:** 2018-08-01

**Authors:** Jinzhu Zhou, Zhiheng Cai, Pengbing Zhao, Baofu Tang

**Affiliations:** 1 Key Laboratory of Electronic Equipment Structure Design, Ministry of Education, Xidian University, Xi’an 710071, China; jzzhou@xidian.edu.cn (J.Z.); Arnold_czh@126.com (Z.C.); 2 Nanjing Research Institute of Electronic Technology, Nanjing 210039, China; baofu_tang@126.com

**Keywords:** shape sensing, optimal sensor placement, antenna deformation, Fiber Bragg grating, information redundancy

## Abstract

This paper investigates the problem of an optimal sensor placement for better shape deformation sensing of a new antenna structure with embedded or attached Fiber Bragg grating (FBG) strain sensors. In this paper, the deformation shape of the antenna structure is reconstructed using a strain–displacement transformation, according to the measured discrete strain data from limited FBG strain sensors. Moreover, a two-stage sensor placement method is proposed using a derived relative reconstruction error equation. In this method, the initial sensor locations are determined using the principal component analysis based on orthogonal trigonometric (i.e., QR) decomposition, and then a new location is sequentially added into the initial sensor locations one by one by minimizing the relative reconstruction error considering information redundancy. The numerical simulations are conducted, and the comparisons show that the proposed method is advantageous in terms of the sensor distribution and computational cost. Experimental validation is performed using an antenna experimental platform equipped with an optimal FBG strain sensor configuration, and the reconstruction results show good agreements with those measured directly from displacement sensors. The proposed method has a large potential for the strain sensor placement of complex structures, and the proposed antenna structure with FBG strain sensors can be applied to the future wing-skin antenna or flexible space-based antenna.

## 1. Introduction

With the improvement of the performance requirements of modern antennas, the surface shape of an antenna structure will play an increasing role in guaranteeing the safe and reliable operation of a large antenna structure such as space antenna reflectors [1], aircraft-wing-skin antenna [2], space-based phased array antenna [3], etc. In particular, in a flexible antenna structure, the structural deformations caused by external loads not only influence the structural health, but also deteriorate the antenna electrical performance. Therefore, the deformation monitoring has become an important research area in structural health monitoring and structural control. However, the direct measurement of displacements is often difficult due to the operating conditions. In these cases, it is helpful to use embedded or attached FBG strain sensors to indirectly estimate the shape (or displacement response) of a deformed antenna structure [4].

Several methods have been proposed to estimate the deformed displacements or shapes by utilizing the measured strains from FBG strain sensors. Geometry interpolation method fits experimentally measured strains into an a priori set of global and piece-wise continuous basis functions, and then the displacement field was evaluated [5,6,7,8]. Geometry interpolation method is very effective for the reconstruction of one-dimensional deformations. However, several strain sensors [9] or in situ calibrations [8] are needed for the reconstruction of complex deformations. An inverse finite element method developed by Tessler and Spangler [10] was applied to estimate the deformation field of the plate, shell elements and frame structures [9,11,12]. However, the geometric structure is usually complex in practice, and it is difficult to accurately reconstruct the displacements by using the inverse finite element method. The reconstruction method based on non-uniform strain spectrum can reconstruct non-uniform strain profiles along the grating [13] or determine periodic surface profile oscillation defects of steel materials [14]. Because of considering non-uniform strains along the Bragg gratings, the method can obtain an accurate strain distribution, which can further improve the accuracy of the deformation reconstruction. Many other studies rely on a modal-based algorithm that uses the mode shapes of the structure to transform the measured strains into displacements [15,16], and the method is successfully applied to reconstruct the structural deformation of a two-dimensional composite structure [17] and rotating structure (helicopter rotors and wind turbine blades) [18]. Compared with other methods, the modal method can deal with the boundary conditions and structural topology of complex geometric structure. Therefore, the paper applies the modal method to investigate the shape deformation sensing problem of the antenna structures with embedded or attached FBG sensors.

The reconstructed responses strongly depend on the quality of the measured data, which further depends on the sensor placement. Many authors have researched the sensor placement technique. These techniques usually include the effective independence (EFI) [19], energy-based approach [20], information entropy method [21,22,23], intelligent optimization method [3,24,25,26,27], and so on. The EFI method proposed by Kammer maximizes the Fisher information matrix [19], and the number of sensors is reduced in an iterative way by deleting the degrees of freedoms (dofs) from the mode shapes, which contributes less to mode-shape independence. The energy-based approach utilizes the principle that the distribution of kinetic energy into a mode of a structure depends on the contribution of this mode to the structural response, and the sensor configuration that maximizes the kinetic energy is selected [20]. Information entropy is considered to be a criterion to identify optimal sensor locations, and the optimal sensor configuration is selected as the one that minimizes the information entropy measure since it is a direct measure of the uncertainty in the model parameter estimates [21,22,23]. Combinatorial optimization methods such as genetic algorithm [3,24], colony algorithm [26], and particle swarm algorithm [27] were used to solve the sensor placement problem based on the Fisher information matrix or the modal assurance criterion (MAC). However, all the methods described above are developed for model updating, structural health monitoring, and modal identification, and they are not suitable for the sensor placement of response reconstructions.

In recent years, the sensor placement for response reconstructions has begun to gain the attention. The estimation error minimization (EEM) is a common criterion to obtaining optimal sensor configurations for response reconstructions. In [28], the EEM criterion is presented to determine the number and locations of sensors, and the results indicate that the optimal sensor configuration obtained from the EEM method can provide a more accurate estimation of the entire structure response. According to the EEM principle, Zhang et al. proposed a dual-type sensor placement for multi-scale response reconstruction [29,30]. In their study, the optimization procedure was implemented by deleting the sensor location from the measurable location set one by one, producing the least response reconstruction error. This procedure is easily implemented for simple structures, but when the measurable location set is large, in particular when the optimal sensor number is small compared with the size of the measurable location set, the entire procedure is computationally intensive and time-consuming [31]. In [31,32], sensor placement methods were proposed for dynamic response reconstructions. In the method, the initial sensor locations are determined using the exhaustive search [31] or the rank of the Markov parameter matrix [32], and then a heuristic forward sequential sensor placement algorithm are proposed to add one more sensor to the initial optimal sensor locations. However, the methods do not consider sensor aggregation phenomenon [33,34], which makes the new added sensor possible as a redundant sensor, not only contributing little to the structural reconstruction accuracy, but also resulting in difficulties in the actual sensors installation due to the concentration of sensor positions. In addition, an exhaustive search in evaluating the performance of all possible sensor configurations is computationally prohibitive in practical circumstances.

The objective of this investigation is to develop an optimal sensor placement method for better shape deformation sensing of an antenna structure with FBG strain sensors. This investigation is essential as a demonstration of feasibility toward future applications such as wing-skin antennas or flexible space-based antennas. In the applications, the FBG strain sensors are embedded or attached in the antenna structure, and the measured strains from limited FBG strain sensors are detected to realize the real-time deformation monitoring or electrical compensation. The contributions of this paper are summarized as follows. (1) A new antenna structure with embedded or attached FBG strain sensors is proposed for the first time in this paper. The antenna structure with FBG strain sensors becomes a smart antenna structure, which can provide the shape deformation sensing and structural health monitoring functions, in addition to the electromagnetic receiving-sending and mechanical load-bearing function of the existing antenna. Moreover, a strain–displacement transformation is presented to indirectly estimate the shape deformation, and the relative reconstruction error equation is derived to facilitate optimal sensor placements; (2) A new two-stage sensor placement method considering information redundancy is proposed to determine optimal FBG strain sensor locations for better shape deformation sensing. In this method, the initial sensor locations are determined using the principal component analysis based on QR decomposition, and then a sequential sensor placement algorithm is implemented by adding a sensor location into the initial sensor locations one by one. The comparisons of numerical investigations demonstrate that the proposed sensor placement method can overcome the concentrative problem of sensor locations without reducing the reconstruction accuracy, and that the calculation time of the proposed method is approximately 100 times less than the ones of existing methods.

The remainder of this paper is organized as follows. Section 2 presents the problem statements Section 3 gives the principle of the deformation sensing. Section 4 proposes a two-stage sensor placement method. Numerical investigation is conducted in Section 5. Section 6 presents the measured results of an antenna platform with an optimal FBG sensor configuration. Finally, Section 7 concludes the paper.

## 2. Problem Statements

**Figure 1:**
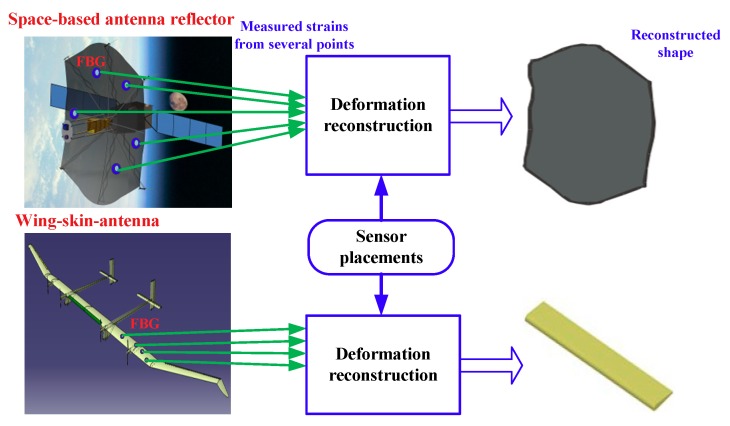
Schematic diagram of the shape deformation sensing based on FBG strain sensors.

The performance requirements of modern airborne and spaceborne antennas are continuously increasing. As a primary load-carrying component, the deformation of antenna structures caused by external loads in service degrades antenna radiation patterns (i.e., electrical performance) [35,36,37]. However, the existing antenna structure cannot preserve the radiation patterns as the changes of the antenna shape, due to the lack of the adaptive shape deformation sensing and radiation-pattern-correction function. Typical measurements for deformation shapes are laser holography and photogrammetry, but they cannot be applied to real-time measurements during operation of some structures such as wing-skin antenna because it is not possible to install the measurement systems somewhere outside the structures. Therefore, this paper proposes a new antenna structure with embedded or attached FBG strain sensors, as shown in Figure 1. Using measured discrete strain data, the deformation shape is estimated using a strain–displacement transformation. Figure 1 shows the concept of the proposed shape deformation sensing method based on attached or embedded FBG strain sensors.

The locations of FBG strain sensors in the antenna structure influence the shape deformation sensing. In practice, considering the high cost of FBG strain sensors and the inaccessibility of some locations, the measured strains are often recorded in a limited number of locations which are much less than the total number of degrees of freedoms (dofs) of the structure. To reconstruct the deformation at unmeasured locations from limited FBG measured strains, a reconstruction algorithm to transform the discrete strains into global displacements is indispensable. Therefore, this paper will study the following two problems. One is how to realize the shape deformation sensing using limited FBG strain sensors which do not locate in all locations of the antenna structure; the other is how to determine an optimal FBG sensor placement for a better deformation reconstruction.

## 3. Deformation Reconstruction

In this section, the strain–displacement transformation is presented to realize the shape sensing of a deformed antenna structure, and then a relative reconstruction error equation is derived to facilitate the following investigation of the optimal sensor placement.

### 3.1. Strain–Displacement Transformation

The measurement principle of the FBG strain sensors is based on the detection of the back-reflected wavelength shift produced in an optical fiber. According to the grating theory [17,18], if there is no temperature change and non-uniform strains along the Bragg gratings, the structural strain ε can be simply expressed as:

(1) $$\varepsilon =\frac{1}{1-p_{e}}\frac{\Delta \lambda_{B}}{\lambda_{B}}\;$$

where $p_{e}$ is the photoelastic constant of an optical fiber, and it is usually 0.22 for a germanosilicate glass, $\lambda_{B}$ and $\Delta \lambda_{B}$ are the Bragg wavelength and wavelength shift, respectively.

Utilizing the modal approach [17,18], the displacement and strain of an antenna structure are expressed as:

(2) $$q=\Phi q_{m}$$

(3) $$\varepsilon =\Psi q_{m}$$

where $q\in R^{N\times 1}$, $\varepsilon \in R^{N\times 1}$, $\Phi \in R^{N\times m}$, $\Psi \in R^{N\times m}$, $q_{m}\in R^{m\times 1}$ represent the displacement, strain, candidate displacement mode shapes, candidate strain mode shapes and modal coordinates, respectively.N and m denotes the number of dofs and the number of used modes, respectively.

Suppose that M FBG strain sensors are embedded into the structural surface and that the position of each FBG sensor is expressed as $d={\left[x,y,z\right]}^{T}$.Therefore, according to (3), the measured strains at the M locations are expressed as:

(4) $$\varepsilon_{M}=\Psi_{M}\left(d\right)q_{m}\;$$

where $\varepsilon_{M}\in R^{M\times 1}$ is the measured strains, $\Psi_{M}\left(d\right)\in R^{M\times m}$ denotes the measured strain mode shapes determined by the sensor locations which are chosen from the N dofs (M≤N).

When the number of measured strains is great than or equal to the number of used modes, i.e., M≥m, the method of least squares approximation can be applied to obtain the modal coordinates:

(5) $$q_{m}^{}={\left(\Psi_{M}^{T}\left(d\right)\Psi_{M}\left(d\right)\right)}^{-1}\Psi_{M}^{T}\left(d\right)\varepsilon_{M}=\Psi_{M}^{+}\left(d\right)\varepsilon_{M}\;$$

where $\Psi_{M}^{+}\left(d\right)\in R^{m\times M}$ denotes the pseudo-verse of the measured strain mode shapes.

According to (2), the reconstructed displacements are expressed as:

(6) $$\hat{q}=\Phi_{s}\Psi_{M}^{+}\left(d\right)\varepsilon_{M}=T\left(d\right)\varepsilon_{M}\;$$

where $\hat{q}\in R^{n\times 1}$ is the reconstructed displacement vector of n pivotal locations. $\Phi_{s}\in R^{n\times m}$ are the measured displacement mode shapes of n interested positions in the structure, and $T\left(d\right)\in R^{n\times M}$ is a transformation matrix between estimated displacements and measured strains.

An antenna structure belongs to a two-dimensional structure, and there are three strain model shapes: $\varepsilon_{Mx}$ and $\varepsilon_{My}$ for the principal strain and $\gamma_{Mxy}$ for the shear. To use (6), the strains in any arbitrary direction can be calculated using the following equation:

(7) $$\varepsilon_{M}=\frac{1}{2}\left(\varepsilon_{Mx}+\varepsilon_{My}\right)+\frac{1}{2}\left(\varepsilon_{Mx}-\varepsilon_{My}\right)\cos2\alpha +\frac{1}{2}\gamma_{Mxy}\sin2\alpha \;$$

where α is the angle between the principal axis and the arbitrary measurement direction. In this work, the FBG strain sensors are pasted on the antenna surface in an orthogonal arrangement at each location. Therefore, the angle is equal to 90 degrees.

Equation (6) is applied to estimate the displacements at the unmeasured locations from limited measured strains. The transformation matrix T(d) can be obtained through the modal analysis. Therefore, if the strains of the measured points are known, the displacements can be reconstructed.

### 3.2. Relative Reconstruction Error Equation

In practice, the measured strain $\varepsilon_{M}$ is usually polluted with measurement noise denoted as e. Considering the effect of the noise, the practical measured strain is written as:

(8) $$\varepsilon_{M}=\Psi_{M}\left(d\right)q_{m}+e\;$$

where e is assumed to be zero mean Gaussian noise with a variance of $\sigma_{}^{2}$.

Utilizing (6) and (2), the reconstruction errors δ(d) are expressed as:

(9) $$\delta \left(d\right)=\hat{q}-q=\Phi_{s}\Psi_{M}^{+}\left(d\right)\left(\Psi_{M}\left(d\right)q_{m}+e\right)-\Phi_{s}q_{m}\;=T\left(d\right)e$$

Subsequently, the covariance matrix of the reconstruction error can be calculated as:

(10) $$\Delta \left(d\right)=E\left(\delta \left(d\right)\delta^{T}\left(d\right)\right)=T\left(d\right)E\left(ee_{}^{T}\right)T^{T}\left(d\right)\;$$

where $E\left(ee_{}^{T}\right)$ is the covariance matrix of the measurement noise.

Because the measurement noise is a zero-mean stationary Gaussian noise and uncorrelated with each other, $E\left(ee_{}^{T}\right)$ is also written as:

(11) $$E\left(ee^{T}\right)=diag\left(\sigma_{}^{2}\right)\;$$

Substituting (11) into (10), the covariance matrix of the reconstruction error is written as:

(12) $$\Delta \left(d\right)=\left[T\left(d\right)diag\left(\sigma_{}^{2}\right)\right]{\left[T\left(d\right)diag\left(\sigma_{}^{2}\right)\right]}^{T}=\sigma^{2}T\left(d\right)T^{T}\left(d\right)\;$$

The measurement noise does not influence the sensor positions. Therefore, this paper introduces a relative reconstruction error equation $\tilde{\Delta }\left(d\right)$, which is the ratio of the covariance matrix of the reconstruction error to the measurement noise.

(13) $$\tilde{\Delta }\left(d\right)=T\left(d\right)T^{T}\left(d\right)\;$$

## 4. Two-Stage Sensor Placement

**Figure 2:**
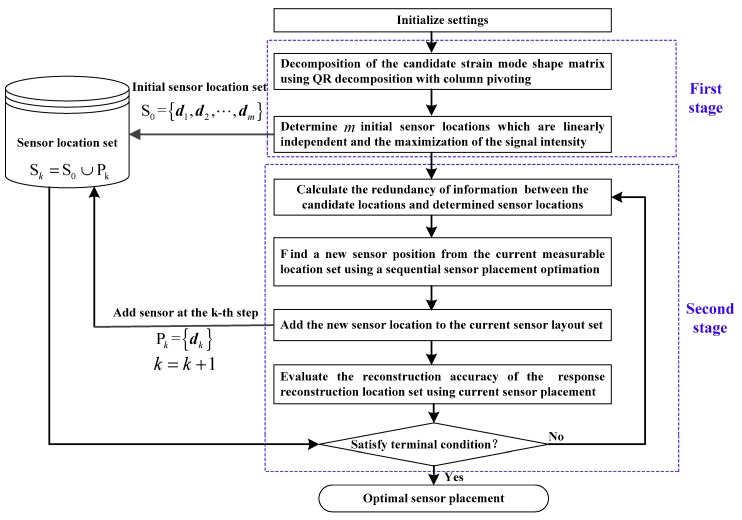
Flow chart of two-stage sensor placement method.

The shape reconstruction based on Equation (6) is influenced by the FBG strain sensor placements. In this work, the FBG strain sensors are pasted on the surface of the antenna structure in an orthogonal arrangement at each location, as suggested in [17]. Therefore, the optimization of the FBG strain sensors locations becomes a critical issue for structural shape reconstruction. Utilizing the strain–displacement relationship (6) and the relative reconstruction error Equation (13), this section proposes a two-stage sensor placement method for better shape deformation sensing. Figure 2 shows the proposed two-stage sensor placement method.

According to Figure 2, initializations should be firstly conducted. The initializations include the determination of the mode number m, the allowed total sensor number M and maximum reconstruction error $e_{\max}^{}$, according to the reconstruction requirements. In addition, the response reconstruction location set $\Gamma_{rrs}$ and the measurable location set $\Gamma_{feas}$ should be determined. As for the antenna structure, the response reconstruction location set is the collection of the central positions of all antenna elements, which influences the antenna electrical performance. The measurable location set is the collection of those locations where the sensors can be installed, and the strain response can be measured. The measurable location set should be determined based on the consideration of some practical issues. For example, some locations where sensors cannot be installed are also not considered as the candidates. Subsequently, the initial sensor location set is chosen from the measurable location set at the first stage. In this paper, the initial sensor location set is determined using QR decomposition of the candidate strain mode-shape matrix. Finally, a sequential sensor placement algorithm is implemented at the second stage by adding a new sensor location into the initial sensor configuration set one by one within the measurable locating set. Combining the initial sensor locations and the added sensor locations, the final sensor placement scheme can be obtained. In the following, the proposed method is explained in detail.

### 4.1. Initial Sensor Placement at the First Stage

**Figure 3:**
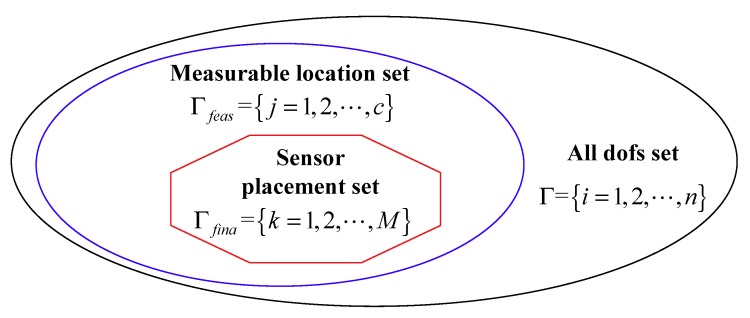
Relationship of sensor layout set, measurable location set and all dofs set.

As for the response reconstruction location set in a definite structure, the displacement mode shape $\Phi_{s}$ in Equation (6) is also a definite matrix. Therefore, the reconstructed response is primarily influenced by the pseudo-verse $\Psi_{M}^{+}\left(d\right)$ and the measured strains $\varepsilon_{M}\left(t\right)$. However, only the pseudo-verse $\Psi_{M}^{+}\left(d\right)$ is related to the sensor positions d. From Equation (4), it is observed that $\Psi_{M}\left(d\right)\in R^{M\times m}$ is a subset of the candidate (or measurable) strain mode-shape matrix $\Psi_{c}\left(d\right)\in R^{c\times m}$, if M FBG sensor locations are chosen from the measurable location set $\Gamma_{feas}\;$. The modal coordinates $q_{m}^{}\left(t\right)$ in Equation (5) is the least squares estimation of Equation (4), which means that Equation (4) is an overdetermined equation. When it satisfies the condition M≥m, one can obtain a unique solution of the modal coordinate. Therefore, as for Equation (4), it requires at least m FBG strain sensors to reconstruct the displacement response. In this subsection, the m initial sensors are determined using the principal component analysis based on QR decomposition. Figure 3 shows the relationship between the sensor placement set, measurable location set and all dofs set. It is observed that the sensor layout set is a subset of the measurable location set, and that it satisfies the condition n≥c≥M≥m.

Suppose that the strains $\varepsilon_{c}\left(t\right)\in R^{m\times 1}$ are measured by m FBG strain sensors. This problem is to find a sensor placement set $S_{0}=\left\{d_{1},d_{2},\cdots ,d_{m}\right\}$ from the measurable location set $\Gamma_{feas}\;$, and make the estimated strains of the chosen location set $S_{0}$ approximate the measured ones. The problem is expressed as:

(14) $$\begin{array}{l} \mathrm{Find}:\;S_{0}=\left\{d_{1},d_{2},\cdots ,d_{m}\right\}\\ \mathrm{Min}:{\left\|\Psi_{m}\left(d\right)q_{m}\left(t\right)-\varepsilon_{c}\left(t\right)\right\|}_{2}^{2}\\ s.t.\;d\in \Gamma_{feas},\Psi_{m}\left(d\right)\in \Psi_{c}\left(d\right) \end{array}\;$$

where the matrix $\Psi_{m}\left(d\right)\in R^{m\times m}$ is chosen from the candidate strain mode-shape matrix $\Psi_{c}\left(d\right)\in R^{c\times m}$ and it depends on the chosen sensor placement location set $S_{0}=\left\{d_{1},d_{2},\cdots ,d_{m}\right\}$.

In this paper, QR decomposition with column pivoting is applied to choose the m rows from the transpose of the matrix $\Psi_{c}\left(d\right)$. In the sense of mathematics, QR decomposition with column pivoting is derived from the maximization of the signal intensity, and the linearly independent row vectors are extracted from the matrix $\Psi_{c}^{T}\left(d\right)$ [38]. The transpose is taken, because the information for one given degree of freedom is provided in one single row of the modal matrix and the QR decomposition does only sort the columns and not the rows of a matrix. The degrees of freedom corresponding with the first m linearly independent row vectors can be used as an initial placement for FBG strain sensors. The decomposition can be expressed as:

(15) $$\Psi_{c}^{T}\left(d\right)E=QR\;$$

where E is the unit conversion matrix, descending order according to the value of the diagonal elements of R. We choose the modal dofs in accordance with the sequence of the elements in E.

According to the principal component analysis based on QR decomposition [39], the remaining (c−m) locations are highly correlated with the initial sensor locations. It should be noted that the error of the reconstructed responses from the initial sensor placement set is small and acceptable. If the economic and technical conditions permit it in practice, additional sensor locations can be included to the initial sensor locations through the following sequential sensor placement to obtain more accurate reconstructed results.

### 4.2. Sequential Sensor Placement at the Second Stage

At the second stage, the remaining (c−m) locations in the measurable location set are evaluated by adding one sensor to the initial sensor combination at a time, which results in a minimization of the response reconstruction error. From Equation (13), it is observed that each diagonal element in the matrix $\tilde{\Delta }\left(d\right)$ represents the variance of the estimation error for displacement responses. Therefore, to achieve the minimum response reconstruction error, the sequential sensor placement can be performed with the objective to minimize the sum of the reconstruction error:

(16) $$d=\underset{d\in \Gamma^{\prime }{}_{feas}}{\min}\left[trace\left(\tilde{\Delta }\left(d\right)\right)\right]\;$$

where the trace of the matrix $\tilde{\Delta }\left(d\right)$ represents the sum of estimation errors at the response reconstruction location set, and $\Gamma^{\prime }{}_{feas}$ denotes the dynamic measurable location set.

The maximum reconstruction error of the antenna surface influences the antenna electrical performance. Therefore, the maximum reconstruction error is used as a constraint function of the sensor location selection, and it is expressed as:

(17) $$\sigma_{\max}^{2}=\max\left(diag\left(\tilde{\Delta }\left(d\right)\right)\right)$$

Although the minimization of Equation (16) is necessary for obtaining a good reconstruction of the displacement responses, it does not take into account that two dofs can be spatially correlated. The spatial correlation can be caused by the mesh refinement of a finite element model (FEM) [33,34,40]. The FEM with a refined mesh will help an accurate response reconstruction. However, the refinement of a mesh can lead to the drawbacks of spatial correlation and the clustering of sensor locations [40]. For example, let us consider an FBG sensor numbered 1 located on a node 1 and an FBG sensor numbered 2 located on a node 2; two nodes in the refined mesh model can have a major impact individually, but if their information matrices are very close, selecting both may be similar to selecting a single. In addition, if the distance between the nodes 1 and 2 is so spatially close that this distance exceeds the size of the sensor, it is not feasible to install the sensors in two locations in practice. One way to avoid this problem would be to exchange the mesh for a cruder one. However, a cruder mesh is likely to miss some of the best potential sensor locations. Therefore, the spatial correlation of the sensor locations should be considered. To deal with the problem, the redundancy of information proposed by Stephan was applied to evaluate the spatial correlation between sensor locations [33]. A metric of closeness is applied to quantify the redundancy of information, and it was given by a norm of their differences:

(18) $$R_{kl}\left(d\right)=\frac{\left\|A_{k}\left(d\right)-A_{l}\left(d\right)\right\|}{\left\|A_{k}\left(d\right)\right\|+\left\|A_{l}\left(d\right)\right\|}\;$$

(19) $$A_{i}\left(d\right)=\Psi_{i}^{T}\left(d\right)\Psi_{i}^{}\left(d\right)\;i=k,l$$

where $R_{kl}\left(d\right)$ denotes the redundancy of information between the kth candidate sensor position and lth previously determined sensor position. $R_{kl}\left(d\right)$ reaches its limit 1, if $A_{l}\left(d\right)$ and $A_{k}\left(d\right)$ are orthogonal. On the contrary, $R_{kl}\left(d\right)$ reaches its limit 0, if two sensors bringing the same information have close information matrices. $A_{i}\left(d\right)$ is a m×m symmetric matrix, and it denotes the information matrix of the ith candidate sensor position (i=k,l). $\Psi_{i}^{}\left(d\right)$ is a 1×m vector from the ith-row of the strain mode-shape matrix Ψ.

In this paper, the redundancy of information is used as the constraint. Considering the objective function (16) and these constraints, the sensor placement at the second stage is formulated as:

(20) $$\begin{array}{l} \mathrm{Find}:\;d={\left[x,y,z\right]}^{T}\\ \mathrm{Min}:trace\left(\tilde{\Delta }\left(d\right)\right)\\ s.t.\;\left\{ \begin{array}{l} \sigma_{\max}^{2}\leq e_{\max}^{}\\ R_{kl}\geq R_{0}\\ M_{s}=M-m\\ d\in \Gamma^{\prime }{}_{feas} \end{array}\right. \end{array}\;$$

where $e_{\max}^{}$ is the threshold of the maximum reconstruction error, and its selection is subject to users’ decision with consideration of acceptable error and cost issue. M is the predefined sensor number, $M_{s}$ is the sensor number determined at the second stage. The sensor location at this stage is from the dynamic measurable location set $\Gamma^{\prime }{}_{feas}=\left\{j=1,2,\cdots ,\left(c-m-s_{k}\right)\right\}$, where $s_{k}$ denotes all of the sensor locations determined before the kth iteration at the second stage, c and m denotes all of the measurable position number and the previously determined sensor number at the first stage, respectively. $R_{0}$ is the threshold of the redundancy of information. The threshold value 0.5 is suggested in [33].

### 4.3. Implementation Procedure

Detailed procedure of the proposed two-stage sensor placement method is summarized as follows:

(1) Specify the mode truncation number m, the total sensor number M and the allowed maximum reconstruction error $e_{\max}^{}$. In addition, the measurable location set $\Gamma_{feas}$, the response reconstruction location set $\Gamma_{rrs}$ and the number c of the candidate sensor locations are also determined.
(2) Perform QR decomposition of the matrix $\Psi_{c}^{T}\left(d\right)$ using Equation (15), and select the first m columns. The column numbers with respect to $\Psi_{c}^{T}\left(d\right)$ are extracted from the matrix E, and we choose the m initial sensor locations corresponding to the sequence of the elements with the value 1 in the unit conversion matrix E.
(3) Utilize Equation (18) to calculate the redundancy of information between all of the remaining candidate locations and the previously determined sensor locations.
(4) Select an optimal sensor location satisfying Equation (20) from the current measurable location set.
(5) Add the newly selected sensor location to the current sensor placement set $S_{k}$, and update the current measurable location set $\Gamma^{\prime }{}_{feas}$ and the allowable remaining sensor numbers $M_{s}$ in the second stage.
(6) If the termination conditions are satisfied, the program is terminated, otherwise repeat the steps (3)~(6). In this paper, the termination conditions are as follows:
  (a) Whether the requirements of the specified reconstruction accuracy $trace\left(\tilde{\Delta }\left(d\right)\right)$ is satisfied;
  (b) Whether the allowed maximum sensor number M is reached.

## 5. Numerical Experiment

This section presents some numerical investigations of an antenna experimental platform to demonstrate the superiority of the proposed sensor placement method. Moreover, the proposed method was compared with the EEM method [29,30]. Four independent assessment criteria were applied to evaluate the results: spatial distribution of sensor locations, computational time, condition number and reconstruction accuracy.

### 5.1. FEM of the Antenna Experimental Platform

**Figure 4:**
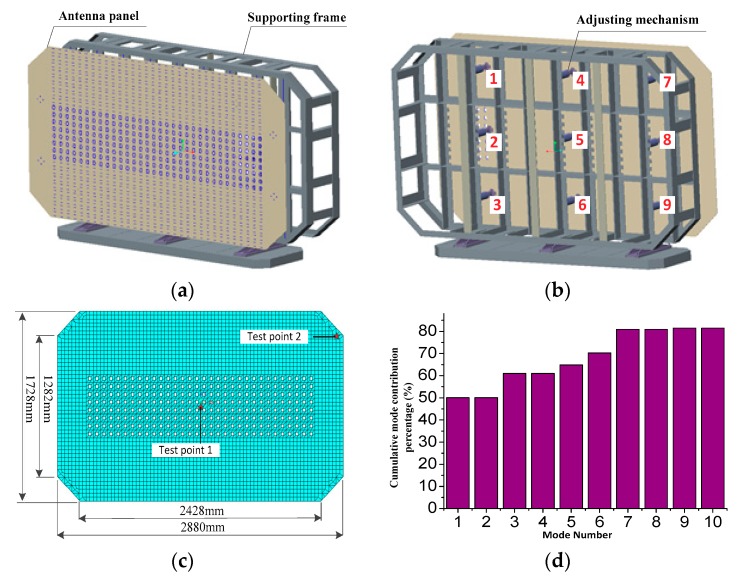
Geometric structure of an antenna experimental platform. (**a**) Front view; (**b**) Rear view; (**c**) Finite element model of the antenna panel; (**d**) Cumulative modal contribution percentage.

The geometric structure of the antenna experimental platform is shown in Figure 4, and it consists of nine adjusting mechanisms, a supporting frame and an antenna panel with FBG strain sensors. The thickness of the antenna panel is 6 mm, and its surface shape is controlled by nine adjusting mechanisms, as shown in Figure 4b. In this numerical experiment, the adjusting mechanisms 1, 2 and 3 were constrained, and the adjusting mechanisms 4, 5, and 6 were removed. The adjusting mechanisms 7, 8 and 9 were applied load to make the panel shape change. The material of the antenna panel is aluminum alloy, and its material parameters are as follows: the modulus of elasticity is 70 GPa, the mass density is 10,044 Kg/m^3^, and the Poisson’s ratio is 0.3. The FEM of the antenna panel was developed by the shell 163 element in ANSYS. Figure 4c shows the FEM. There are 3379 nodes as the candidates for sensor locations, and the distance between two nodes is 36 mm which is about twice the length of the FBG sensor.

After performing the modal analysis, Figure 4d presents the first ten modal contribution of the antenna panel. It is found that the cumulative effective modal mass participation ratio of the first seven modes is 81.7%, and that the contribution of the last three modal is very small. Thus, the first seven modes were selected as the main contribution modes. Table 1 presents the first seven natural frequencies of the antenna panel.

### 5.2. Superiority of Sensor Placement Method

**Figure 5:**
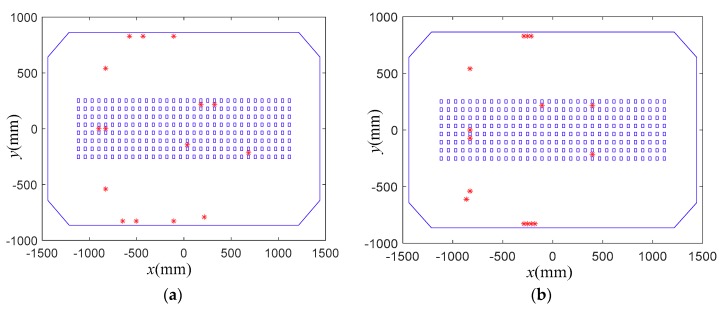
Comparisons of sensor placement scheme for 15 sensor locations. (**a**) Proposed method; (**b**) EEM.

**Figure 6:**
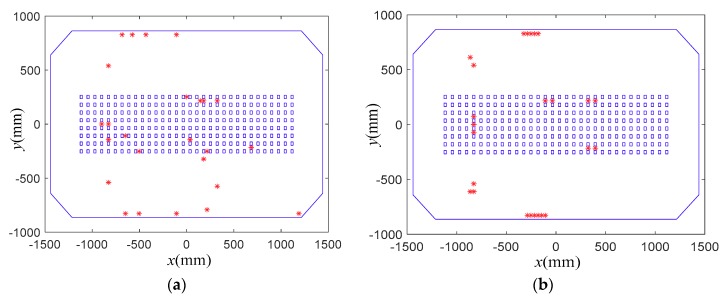
Comparisons of sensor placement scheme for 25 sensor locations. (**a**) Proposed method; (**b**) EEM.

**Figure 7:**
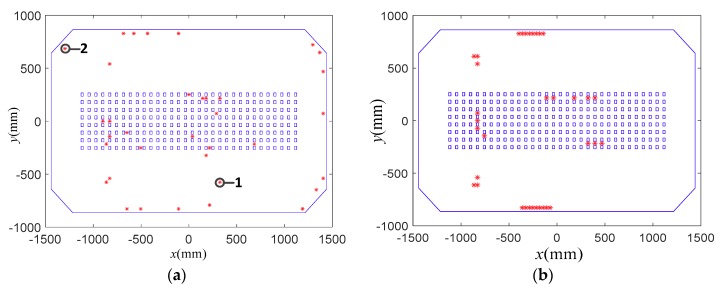
Comparisons of sensor placement scheme for 35 sensor locations. (**a**) Proposed method; (**b**) EEM.

In this subsection, the proposed sensor placement method was applied to determine the FBG strain sensor locations, and the results were compared with those obtained by the EEM method in [29,30]. Figure 5, Figure 6 and Figure 7 present the comparisons of sensor placement schemes obtained by the proposed method and EEM, when the number of the used sensor locations is 15, 25 and 35, respectively.

From the comparisons, it is observed that the spatial distribution of the FBG strain sensor locations obtained by the EEM method is very concentrative. Moreover, with the increase of the sensor number, the concentrative phenomenon of the sensor configurations is more serious. However, the sensor configurations obtained by the proposed method feature a more homogeneous distribution over the whole structure. The reason is that the EEM method does not consider the redundancy of information shared by two dofs. From a practical point of view, it is difficult to install the FBG strain sensors in a very close spatial distribution. The comparisons also show that the proposed method can provide a sensor scheme with a focus on the strong modal response while simultaneously considering the spatial distribution of all sensors.

**Figure 8:**
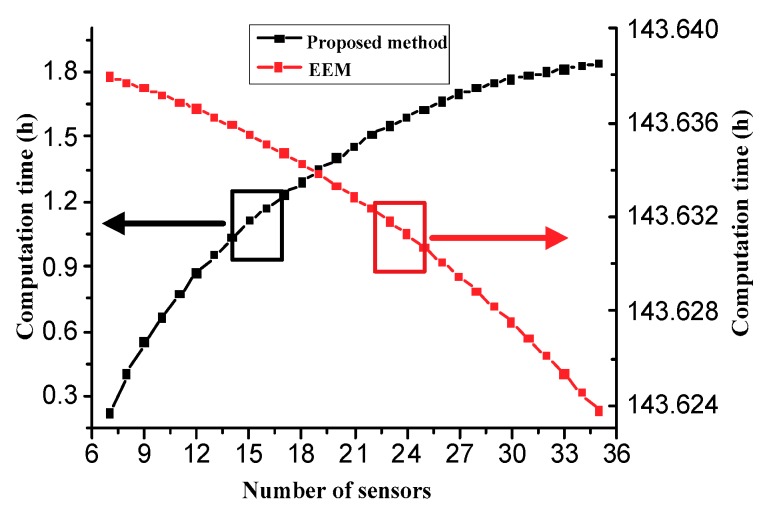
Comparisons of computation time.

Figure 8 shows the calculation time changes with the increase of the number of sensor locations. In the case of the same computer, the calculation time of the sensor placement spent by the proposed method is about 100 times less than the one of the existing method. In addition, the lower the number of the sensor locations, the shorter time spent by the proposed method, and the EEM method is the opposite. Because the number of the sensor placement is much smaller than the number of candidate nodes, the two-stage sensor placement method can obtain higher computational efficiency, and it is very suitable to handle complex FEMs with fine meshes.

The superiority of the proposed method is further validated using the condition number of the transformation matrix. Mathematically, the condition number is described as the ratio between the largest and the smallest singular value of the matrix, and it is defined as:

(21) $$cond\left(T\left(d\right)\right)=\left\|T\left(d\right)\right\|\left\|T{\left(d\right)}^{-1}\right\|\;$$

where $\left\|T{\left(d\right)}^{-1}\right\|$ is the norm of the inverse of the transformation matrix T(d). Large condition numbers indicate a poor choice of sensor configuration. A condition number of 1 indicates that mode-shape matrix is orthogonal, and can obtain robust displacement response reconstruction [17].

**Figure 9:**
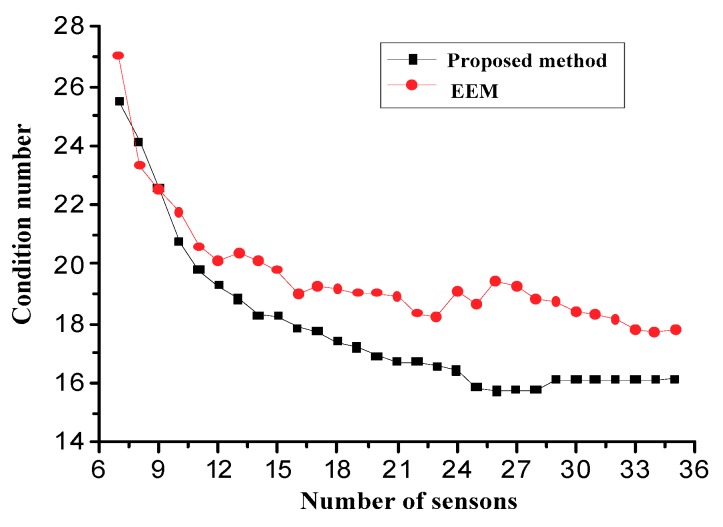
Comparisons of condition numbers.

Figure 9 shows the comparison of the condition numbers of the conversion matrix. From Figure 9, it is found that the condition numbers of the two methods decrease with the increase of the number of sensors. However, when the number of sensors reaches a stable value, the condition number of the proposed method is much smaller than that of the EEM, which indicates that the displacement response reconstruction from the proposed method is superior to the one of the EEM method for noise resistance.

The sensor placement scheme in Figure 7 was applied to evaluate the accuracy of the shape deformation sensing. To include noise corruption, the normally distributed random noise is added to the pollution-free strain response as:

(22) $$\varepsilon^{n}\left(t\right)=\varepsilon +Amp\times randn$$

where $\varepsilon^{n}\left(t\right)$ and ε are the noise-polluted response and pollution-free strain response, respectively; Amp is the noise amplitude, and it is assigned 0.1 ε (10% noise) for the strain gauge in this simulation, and *randn* is a standard normal distribution vector with zero mean and unit standard deviation.

**Figure 10:**
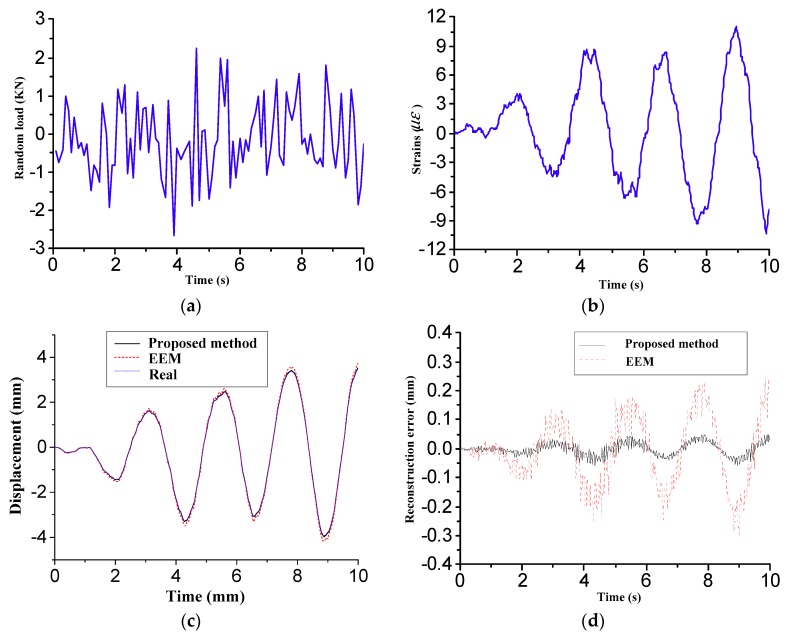
Comparisons of real and reconstructed response time histories. (**a**) External excitations; (**b**) Strain response of FBG strain sensor 1; (**c**) Displacement time history of test point 1; (**d**) Reconstruction errors of test point 1.

Figure 10a shows the external excitations applied on the adjusting mechanisms 7, 8 and 9. Figure 10b shows the measured strains of the FBG strain sensor 1 as shown in Figure 7a. Using Equation (6), the displacement responses of the interested positions in the antenna panel were reconstructed by using the measured strains from the optimal FBG sensor placement shown in Figure 7a. This paper presents the displacement response and reconstruction errors of the test point 1 shown in Figure 4c, due to the limitation of the paper space. Figure 10c,d shows the reconstructed displacement time history and reconstruction errors of the test point 1, respectively. From the comparisons, it is observed that the two methods can well approximate the real response, but that the reconstruction errors of the proposed method are much smaller than the one of the EEM method.

As shown in (22), the random measurement noise affects the reconstruction accuracy. To consider the effect of the random measurement noise, 100 simulation experiments were conducted to investigate the statistical properties of the deformation reconstruction. The noise amplitude Amp in (22) is taken as 0.05ε and 0.25 ε, respectively. The average relative percentage error (ARPE) of the displacement reconstruction is defined as to evaluate the reconstruction accuracy:

(23) $$ARPE=\frac{1}{m}\sum_{j=1}^{m}\frac{\left\|y_{j}\left(t\right)-{\hat{y}}_{j}\left(t\right)\right\|}{\left\|y_{j}\left(t\right)\right\|}\times 100\%$$

where the vector $y_{j}$ and ${\hat{y}}_{j}$ denote the real and estimated displacement of the j-experiment, respectively. The variable m is the number of displacement reconstruction experiment.

**Figure 11:**
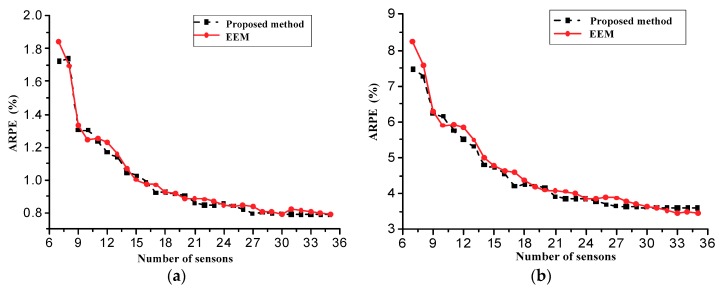
ARPE of displacement reconstructions verses number of sensors. (**a**) 5% noise; (**b**) 25% noise.

Figure 11 presents the ARPE of displacement reconstructions verses the number of sensor locations, when the noise amplitude of the strain measurements is 0.05ε(t) (5% noise) and 0.25ε(t)(25% noise), respectively. From the comparisons, it is observed that the ARPE of the two methods decrease with the increase of the number of sensor locations, and that their reconstruction accuracy improves with the noise reduction. However, the reconstruction accuracy of the proposed method is slightly higher than one of the EEM method.

From the comparisons above, it follows that the superiority of the proposed sensor placement method includes the following several aspects. (1) The proposed method can efficiently avoid the concentrative problem among sensor locations, which facilitates the sensor installation in practice; (2) The calculation time of the proposed method is approximately 100 times less than the ones of the existing methods, and the proposed method is suitable to handle the sensor placements of complex FEMs with thousands of dofs; (3) The reconstruction can well approximate the real response, but that the reconstruction accuracy of the proposed method is slightly higher than the one of the EEM method.

## 6. Experimental Results

This section developed an antenna experimental platform to validate the feasibility toward future applications such as wing-skin antenna or space-based antenna. The effectiveness of the shape deformation sensing and sensor placement method was validated by the experimental platform equipped with an optimal FBG strain sensor configuration.

### 6.1. Experimental System

**Figure 12:**
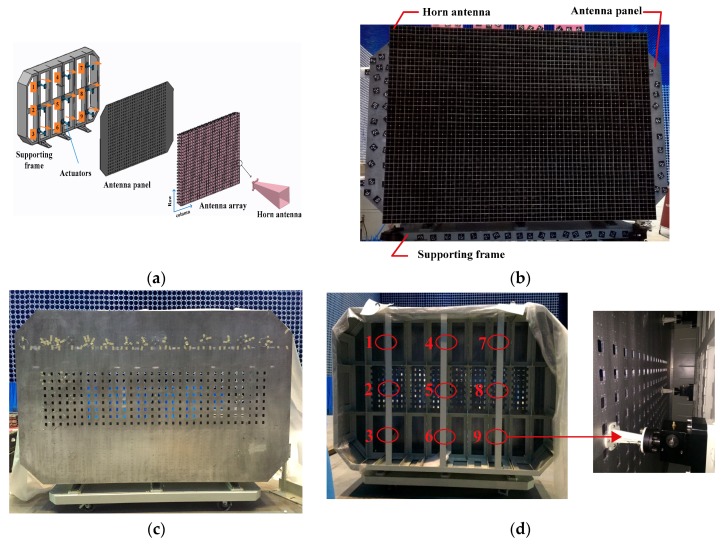
Antenna experimental platform. (**a**) Exploded view of antenna experimental platform; (**b**) antenna experimental platform with 768 horn antenna elements; (**c**) Front view of the antenna panel; (**d**) Nine adjusting mechanisms.

An experimental platform of x-band phased array antenna with 24 × 32 antenna elements was built for the shape deformation sensing experiments, as shown in Figure 12. The experimental platform consists of a supporting frame, nine adjusting mechanisms, and an antenna panel with FBG strain sensors and 768 horn antenna elements. The type of the antenna element is the horn antenna with the operating frequency 10 GHz. The 768 horn antenna elements installed on the surface of the antenna panel were arranged in square grid with the spacing of 19.5 mm. The scanning range of the radiation patterns is from −15° to 15°. The geometrical dimensions and physical properties of the experimental platform are the same as those described in the Section 5.1. The antenna panel and horn antenna element are made of aluminum alloy. In addition, 70 FBG strain sensors in an optimized configuration were attached 35 locations on the surface of the antenna panel, and each two strain sensors were pasted a location in an orthogonal arrangement. The 35 sensor locations determined by the proposed method is given in Figure 7a.

In this experiment, the horn antenna elements were removed to expediently perform the experiments of deformation reconstructions. The shape of the antenna panel was controlled by the adjusting mechanisms shown in Figure 12d. Each adjusting mechanism consists of a torsional stepping motor, a worm gear reducer and a controller for the stepping motor. The excitation signal from the controller makes the stepping motor rotate, and the rotating torque was transformed to a force by using the worm gear reducer. The force was vertically applied at the antenna panel, which leads to the deformation of the antenna panel.

**Figure 13:**
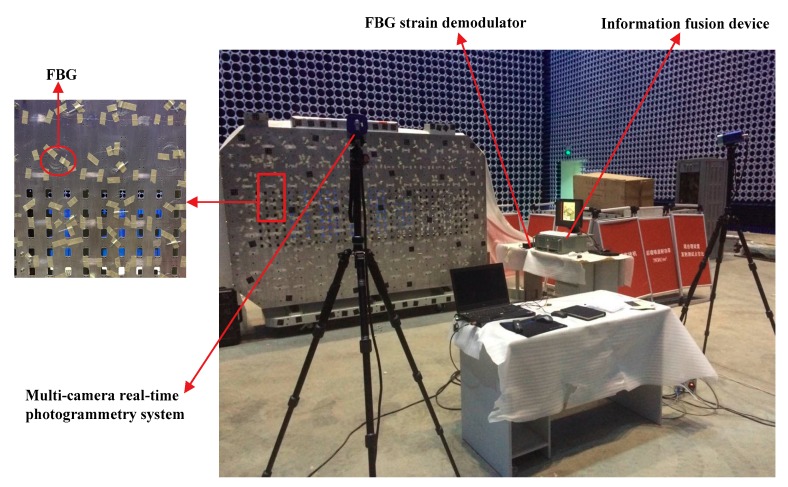
Experimental system for the shape deformation sensing.

The measurement devices consist of an FBG measurement system and a multi-camera real-time photogrammetry system, as shown in Figure 13. The FBG measurement system consists of a commercial FBG demodulator, FBG strain sensors, and a computing and display device. Seven channels of the commercial FBG demodulator (i.e., si255, Micron Optics, Inc., Atlanta, GA, USA) were used to acquire the strain data at a sampling rate of 1000 Hz. If the grating wavelength difference between adjacent FBG sensors is large in the same channel, the interference between the sensors can be reduced. Therefore, ten FBG strain sensors with almost 10 mm grating length were fabricated, and their Bragg wavelengths were 1528 nm, 1534 nm, 1540 nm, 1544 nm, 1548 nm, 1552 nm, 1555 nm, 1558 nm, 1561 nm and 1563 nm, respectively. In addition, the deformations were also measured by a photogrammetry system, which consists of a two digital cameras, photogrammetric basis and target points. All the displacements detected by the target points were employed as the reference displacements to compare with the reconstructed displacements based on the FBG strain sensors. The information fusion device was developed to simultaneously acquire the strains and displacements by controlling the sampling time.

### 6.2. Reconstruction Results

**Figure 14:**
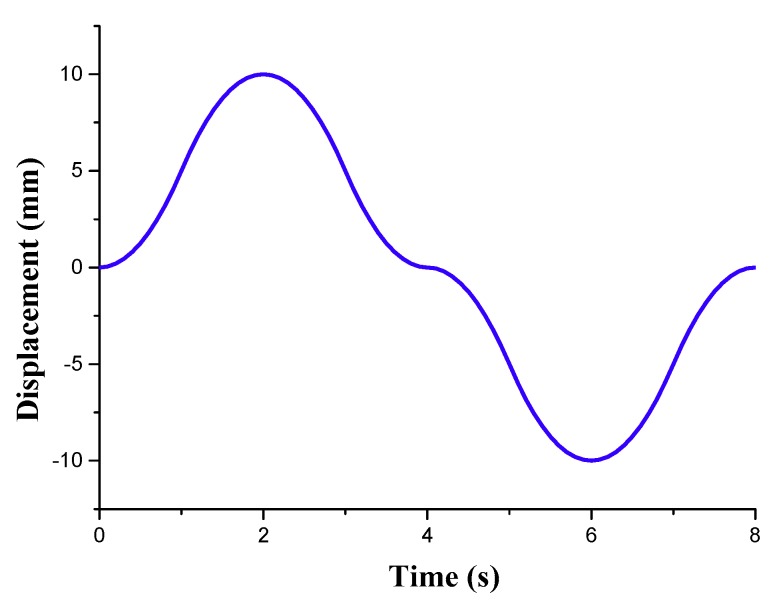
External excitations of the adjusting mechanisms 7, 8 and 9.

This subsection presents some experimental results of the shape deformation sensing. In this experiment. The actuator 1, 2 and 3 were fixed, and the adjusting mechanisms 4, 5, and 6 were removed. The external forces produced by the adjusting mechanisms 7, 8 and 9 were applied vertically at the other end of the antenna panel, which leads to the deformation of the antenna panel. The adjusting mechanisms 7, 8 and 9 were controlled by three torsional stepping motors. Figure 14 presents the displacement excitations of the adjusting mechanisms 7, 8 and 9.

**Figure 15:**
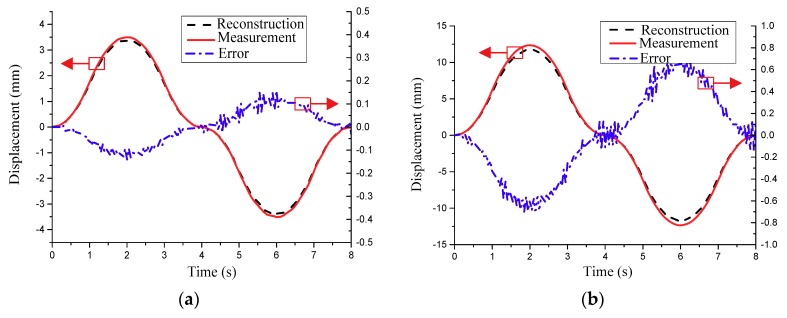
Deformation reconstructions and errors of two test points. (**a**) Test point 1; (**b**) Test point 2.

To compare the reconstructed displacements with the measured displacements, the acquisition of the strain and displacement information should be done at the same time. In this work, the sampling frequency of the strain and displacement was controlled by the information fusion device. Using the limited measured strains from the FBG strain sensors, Equation (6) was utilized to reconstruct the displacement responses of interest positions. Finally, the reconstructed displacements were validated by the measured displacements from the multi-camera real-time photogrammetry system. Figure 15 shows the displacement responses and reconstruction errors of the test points 1 and 2 shown in Figure 4c. From Figure 15, it is observed that the reconstructed displacements agree with the measured ones. Table 2 presents the reconstruction errors under the dynamic deformations. As shown in Table 2, the errors at the test point 2 are bigger that the ones of the test point 1, due to a larger deformation at the test point 2. The reasons for the reconstruction errors in Table 2 could be explained by the following aspects. First, the strain data acquisition system has certain measurement errors, in particular for the large deformations or the dynamic deformations with a high vibration frequency. Second, the transformation matrix in Equation (6) was formed by the displacement and strain mode shapes which are from the FEM of the antenna panel. Any modeling error in the FEM of the antenna panel influenced the mode shapes, which further caused the reconstruction errors. Therefore, the reconstruction errors can be further reduced by using model update to obtain a more accurate finite element model or reducing the measurement noises of the strains.

**Figure 16:**
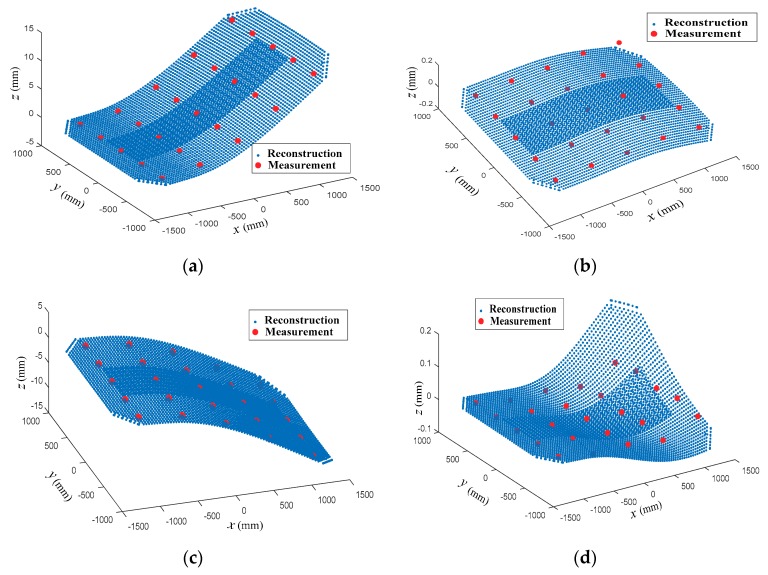
Comparisons of reconstructed and measured shapes. (**a**) Reconstructed shape at 2 s; (**b**) Reconstructed shape at 4 s; (**c**) Reconstructed shape at 6 s; (**d**) Reconstructed shape at 8 s.

Figure 16 shows the whole estimated displacement field of the deformed antenna panel at some moments. In Figure 16, red dots represent the measured displacements using displacement sensors (i.e., target points) of the multi-camera real-time photogrammetry system. From Figure 16, we can see the good agreement between the estimated shapes and the measured data at the target points.

### 6.3. Result Discussions

As presented in the preceding section, the reconstructed shapes based on an optimal FBG strain sensor placement are close to the real ones. Although there are some reconstruction errors, they are acceptable in practice. It follows that it is feasible to monitor the structural deformation by using limited optimal FBG sensor placement. Considering the FBG characteristics such as high precision, light weight, strong anti-electromagnetic interference and easily integration, the antenna structure with optimal FBG sensor placement can be easily fabricated. It is a convenient and economical method for the indirect measurement of structural displacement, because of a small number of discrete strain sensors needed, in particular for situations where direct measurement of structural displacement (e.g., wing-skin antenna) is difficult.

It should be noted that the antenna panel in this experiment has a monolithic nature with uniform material properties and constant thickness, which makes the modeling relatively simple. The errors of deformation reconstructions in Table 2 can be further reduced by using a more accurate finite element model or reducing the strain measurement noises. The model update is a common method to improve the accuracy of the finite element model in practice. However, for a realistic complex structure, achieving a more accurate finite element model that closely matches the real structure might be a daunting task, but can significantly increase the reconstruction accuracy. In addition, the reconstruction results can be also improved by increasing the number of modes and the number of sensors, but which will lead to a greater computational effort.

The FBG strain sensor placement influences the reconstructed shapes, as shown in (6). Therefore, a two-stage sensor placement method was proposed to obtain a better shape deformation sensing. The investigation of the antenna panel validate that the proposed method can obtain a disperse FBG sensor distribution over the whole structure, and it can efficiently avoid the concentrative problem among sensor locations, which facilitates the sensor installation in practice. Moreover, the calculation time of the proposed method is approximately 100 times less than the ones of the existing methods. The results imply that the proposed method can handle the sensor placements of complex FEMs with thousands of dofs.

## 7. Conclusions

A two-stage sensor placement method is proposed for better shape reconstruction at interested locations where no sensors are installed. In this method, the initial sensor locations are determined using the principal component analysis based on the QR decomposition, and then a new location is sequentially added into the initial sensor locations one by one by minimizing the reconstruction errors considering information redundancy. Combining the initial sensor locations and added sensor locations, the final sensor placement scheme can be obtained. Numerical simulations were conducted to illustrate the superiority of the proposed method. The comparisons show that the proposed method can not only improve the reconstruction accuracy, but also avoid the clustering problem among sensor locations. The sensor configurations obtained by the proposed method feature a more homogeneous distribution over the whole structure, which facilitates the installation of sensors in practice. Moreover, the calculation time of the proposed method is approximately 100 times less than the ones of the existing methods. The proposed method was applied to arrange the optimal FBG strain sensor locations in an antenna experimental platform. The shape deformation sensing experiments were conducted to verify the effectiveness and feasibility of the proposed method, and the experimental results demonstrate that the reconstructed shapes agree well with the real ones. The proposed antenna structure with embedded or attached FBG strain sensors is suitable for the development of the adaptive shape monitoring and electrical compensation in these applications such as the smart skin antenna installing into the fuselage and the wings of an aircraft, and space-based flexible antenna. Because of the advantages of the sensor distribution and computational cost, the proposed two-stage sensor placement method has a large application prospect for strain sensor placements of large structures with fine meshes in practice.

## Figures and Tables

**Table 1:** First seven natural frequencies of the antenna panel.

Modal	1	2	3	4	5	6	7
Frequency (Hz)	0.45	1.47	2.78	5.05	6.03	6.40	8.52

**Table 2:** Errors of deformation reconstructions.

Locations	RMSE (mm)	MAE (mm)	ARPE
Test point 1	0.0741	0.1587	3.43%
Test point 2	0.3977	0.7240	5.22%

## References

[1] Wang Z.W., Li T.J. (2014). Optimal piezoelectric sensor/actuator placement of cable net structures using *H*_2_-norm measures. J. Vib. Control.

[2] Hajrya R., Mechbal N. (2013). Principal component analysis and perturbation theory-based robust damage detection of multifunctional aircraft structure. Struct. Health Monit..

[3] Yang C., Zhang X.P., Huang X.Q., Cheng Z.A., Zhang X.H., Hou X.B. (2017). Optimal sensor placement for deployable antenna module health monitoring in ssps using genetic algorithm. Acta Astronaut..

[4] Li N.-L., Jiang S.-F., Wu M.-H., Shen S., Zhang Y. (2018). Deformation monitoring for chinese traditional timber buildings using fiber bragg grating sensor. Sensors.

[5] Zhang H.S., Zhu X.J., Gao Z.Y., Liu K.N., Jiang F. (2016). Fiber bragg grating plate structure shape reconstruction algorithm based on orthogonal curve net. J. Intell. Mater. Syst. Struct..

[6] Yi J.C., Zhu X.J., Zhang H.S., Shen L.Y., Qiao X.P. (2012). Spatial shape reconstruction using orthogonal fiber bragg grating sensor array. Mechatronics.

[7] Derkevorkian A., Masri S.F., Alvarenga J., Boussalis H., Bakalyar J., Richards W.L. (2013). Strain-based deformation shape-estimation algorithm for control and monitoring applications. AIAA J..

[8] Wang Z.F., Wang J., Sui Q.M., Li S.C., Jia L. (2016). In-situ calibrated deformation reconstruction method for fiber bragggrating embedded smart geogrid. Sens. Actuators A Phys..

[9] Kefal A., Yildiz M. (2017). Modeling of sensor placement strategy for shape sensing and structural health monitoring of a wing-shaped sandwich panel using inverse finite element method. Sensors.

[10] Tessler A., Spangler J.L. (2005). A least-squares variational method for full-field reconstruction of elastic deformations in shear-deformable plates and shells. Comp. Methods Appl. Mech. Eng..

[11] Gherlone M., Cerracchio P., Mattone M., Di Sciuva M., Tessler A. (2014). An inverse finite element method for beam shape sensing: Theoretical framework and experimental validation. Smart Mater. Struct..

[12] Gherlone M., Cerracchio P., Mattone M., Di Sciuva M., Tessler A. (2012). Shape sensing of 3D frame structures using an inverse finite element method. Int. J. Solids Struct..

[13] Zheng S.J., Zhang N., Xia Y.J., Wang H.T. (2014). Research on non-uniform strain profile reconstruction along fiber bragg grating via genetic programming algorithm and interrelated experimental verification. Opt. Commun..

[14] Sławomir C., Piotr K. (2016). Inverse problem of determining periodic surface profile oscillation defects of steel materials with a fiber bragg grating sensor. Appl. Opt..

[15] Kang L.H., Kim D.K., Han J.H. (2007). Estimation of dynamic structural displacements using fiber bragg grating strain sensors. J. Sound Vib..

[16] Davis M.A., Kersey A.D., Sirkis J., Friebele E.J. (1996). Shape and vibration mode sensing using a fiber optic bragg grating array. Smart Mater. Struct..

[17] Rapp S., Kang L.H., Han J.H., Mueller U.C., Baier H. (2009). Displacement field estimation for a two-dimensional structure using fiber bragg grating sensors. Smart Mater. Struct..

[18] Kim H.I., Kang L.H., Han J.H. (2011). Shape estimation with distributed fiber bragg grating sensors for rotating structures. Smart Mater Struct.

[19] Kammer D.C. (1991). Sensor placement for on-orbit modal identification and correlation of large space structures. J. Guid. Control Dyn..

[20] Li D.S., Li H.N., Fritzen C.P. (2007). The connection between effective independence and modal kinetic energy methods for sensor placement. J. Sound Vib..

[21] Papadimitriou C., Beck J.L., Au S.K. (2000). Entropy-based optimal sensor location for structural model updating. J. Vib. Control.

[22] Yuen K.V., Kuok S.C. (2015). Efficient bayesian sensor placement algorithm for structural identification: A general approach for multi-type sensory systems. Earthq. Eng. Struct. Dyn..

[23] Bertola N., Papadopoulou M., Vernay D., Smith I. (2017). Optimal multi-type sensor placement for structural identification by static-load testing. Sensors.

[24] Yao. L., Sethares W.A., Kammer. D.C. (1993). Sensor placement for on-orbit modal identification via a genetic algorithm. AIAA J..

[25] Akbarzadeh V., Levesque J.C., Gagne C., Parizeau M. (2014). Efficient sensor placement optimization using gradient descent and probabilistic coverage. Sensors.

[26] Feng S., Jia J.Q. (2016). 3D sensor placement strategy using the full-range pheromone ant colony system. Smart Mater. Struct..

[27] Zhang X., Li J.L., Xing J.C., Wang P., Yang Q.L., Wang R.H., He C. (2014). Optimal sensor placement for latticed shell structure based on an improved particle swarm optimization algorithm. Math. Probl. Eng..

[28] Chen W., Zhao W.G., Zhu H.P., Chen J.F. (2014). Optimal sensor placement for structural response estimation. J. Cent. South Univ..

[29] Zhang X.H., Zhu S., Xu Y.L., Homg X.J. (2011). Integrated optimal placement of displacement transducers and strain gauges for better estimation of structural response. Int. J. Struct. Stab. Dyn..

[30] Zhang X.H., Xu Y.L., Zhu S.Y., Zhan S. (2014). Dual-type sensor placement for multi-scale response reconstruction. Mechatronics.

[31] Zhang C.D., Xu Y.L. (2016). Optimal multi-type sensor placement for response and excitation reconstruction. J. Sound Vib..

[32] Wang J., Law S.S., Yang Q.S. (2014). Sensor placement method for dynamic response reconstruction. J. Sound Vib..

[33] Stephan C. (2012). Sensor placement for modal identification. Mech. Syst. Signal Process..

[34] Lian J.J., He L.J., Ma B., Li H.K., Peng W.X. (2013). Optimal sensor placement for large structures using the nearest neighbour index and a hybrid swarm intelligence algorithm. Smart Mater. Struct..

[35] Zhou J., Huang J., He Q., Tang B., Song L. (2016). Development and coupling analysis of active skin antenna. Smart Mater. Struct..

[36] Zhou J., Song L., Huang J., Wang C. (2015). Performance of structurally integrated antennas subjected to dynamical loads. Int. J. Appl. Electromagn. Mech..

[37] Wang H.S.C. (1992). Performance of phased-array antennas with mechanical errors. IEEE Trans. Aerosp. Electron. Syst..

[38] Zhu L.H., Dai J., Bai G.L. (2015). Sensor placement optimization of vibration test on medium-speed mill. Shock Vib..

[39] Sharma A., Paliwal K.K., Imoto S., Miyano S. (2013). Principal component analysis using QR decomposition. Int. J. Mach. Learn. Cybern..

[40] Friswell M.I., Castro-Triguero R. (2015). Clustering of sensor locations using the effective independence method. AIAA J..

